# Understanding itch in cutaneous T-cell lymphoma: Exploring the impact of comorbidities, treatment regimens, and racial differences on quality of life

**DOI:** 10.1016/j.jdin.2022.11.006

**Published:** 2022-11-23

**Authors:** Tejas P. Joshi, Madeleine Duvic

**Affiliations:** a School of Medicine, Baylor College of Medicine, Houston, Texas; b Department of Dermatology, University of Texas, MD Anderson Cancer Center, Houston, Texas

**Keywords:** comorbidities, cutaneous T-cell lymphoma, folliculotropic mycosis fungoides, mycosis fungoides, quality of life, racial differences

*To the Editor:* We read with interest the paper by Ottevanger et al,^1^ which explores the impact of itch on the quality of life (QoL) of patients with cutaneous T-cell lymphoma (CTCL). The investigators show pruritus to have a significant impact on the QoL of patients with CTCL. Importantly, the authors show more advanced CTCL to be associated with poorer QoL, extending our previous work on this topic.^2^ Here, we wish to point out some limitations of the study and highlight areas for further research.

To adjust for comorbidities as a potential confounder, the authors use the Charlson comorbidity index to quantify the burden of comorbid disease.^1^ Although the Charlson comorbidity index is a validated tool to account for comorbid diseases, it fails to capture important comorbidities present in patients with CTCL. Recently, we have shown mycosis fungoides to be significantly associated with inflammatory bowel disease, hypothyroidism, and vitiligo—important autoimmune illnesses not considered by the Charlson comorbidity index.^3^ Additionally, the authors further characterize the comorbidity burden as low (0-1 comorbidities) and high (≥2 comorbidities)^1^; however, such a stratification fails to account for the fact that certain comorbidities may have a more pronounced impact on QoL. Vitiligo, for instance, is associated with significant psychosocial morbidity, and the presence of vitiligo alone may bear a more negative QoL burden than the sum of other comorbidities.

Another important limitation of the analysis by Ottevanger et al^1^ is that the investigators do not describe which treatments, if any, the patients were undergoing during the study; however, certain treatments for CTCL are known to mitigate pruritis. For example, the histone deacetylase inhibitors romidepsin and vorinostat have been shown to blunt pruritus not just in responders but also in nonresponders. Similarly, patients with folliculotropic mycosis fungoides experience substantial improvement in pruritus after extracorporeal photopheresis and total skin electron beam therapy.^4^ Conversely, pruritus may be an adverse effect of other CTCL treatments—for instance, pruritus has been described as an adverse effect of the immune checkpoint inhibitor nivolumab. Therefore, failing to consider treatment regimens does not afford a clear picture of the precise link between pruritus and QoL.

Finally, the authors do not clarify the ethnic or racial make-up of the study participants. It is important to acknowledge these demographics, as certain populations may experience poorer QoL. Indeed, in a recent study, Abraham et al^5^ showed that skin-of-color patients with CTCL experience poorer QoL compared with their non–skin-of-color counterparts.

There has been a paucity of research investigating the impact of pruritus in CTCL on QoL, and the authors’ effort to characterize the interplay between pruritus and QoL is commendable. However, future studies with more rigorous control for comorbid disease, treatment regimens, and potential ethnic or racial confounders are needed.

## Conflict of interest

None disclosed.
